# Gut Microbiota Metabolism of Bile Acids Could Contribute to the Bariatric Surgery Improvements in Extreme Obesity

**DOI:** 10.3390/metabo11110733

**Published:** 2021-10-27

**Authors:** Luis Ocaña-Wilhelmi, Gracia María Martín-Núñez, Patricia Ruiz-Limón, Juan Alcaide, Eduardo García-Fuentes, Carolina Gutiérrez-Repiso, Francisco J. Tinahones, Isabel Moreno-Indias

**Affiliations:** 1 Departamento de Especialidades Quirúrgicas, Bioquímica e Inmunología, Universidad de Málaga, 29010 Málaga, Spain; luisowilhelmi@hotmail.com; 2 Unidad de Gestión Clínica de Cirugía General y del Aparato Digestivo, Hospital Universitario Virgen de la Victoria, 29010 Málaga, Spain; 3 Department of Endocrinology and Nutrition, Virgen de la Victoria Hospital, Instituto de Investigación Biomédica de Málaga (IBIMA), Málaga University, 29010 Málaga, Spain; graciamaria_mn@hotmail.com (G.M.M.-N.); patriciaruizlimon@ibima.eu (P.R.-L.); juan.alcaidetorres@gmail.com (J.A.); 4 Centro de Investigación Biomédica en Red de la Fisiopatología de la Obesidad y Nutrición (CIBEROBN), Instituto de Salud Carlos III (ISCIII), 29029 Madrid, Spain; 5 Department of Gastroenterology, Virgen de la Victoria University Hospital, Institute of Biomedical Research in Málaga (IBIMA), Málaga University, 29010 Málaga, Spain; edugf1@gmail.com; 6 CIBER Enfermedades Hepáticas y Digestivas-CIBEREHD, Instituto de Salud Carlos III, 28029 Madrid, Spain

**Keywords:** bile acids, bariatric surgery, gut microbiota, Enterobacteriaceae

## Abstract

Bariatric surgery is the only procedure to obtain and maintain weight loss in the long term, although the mechanisms driving these benefits are not completely understood. In the last years, gut microbiota has emerged as one of the drivers through its metabolites, especially secondary bile acids. In the current study, we have compared the gut microbiota and the bile acid pool, as well as anthropometric and biochemical parameters, of patient with morbid obesity who underwent bariatric surgery by two different techniques, namely Roux-en-Y gastric bypass (RYGB) or sleeve gastrectomy (SG). Gut microbiota populations differed after the respective procedures, particularly with respect to the *Enterobacteriaceae* family. Both techniques resulted in changes in the bile acids pool, but RYGB was the procedure which suffered the greatest changes, with a reduction in most of their levels. *Blautia* and *Veillonella* were the two genera that more relationships showed with secondary bile acids, indicating a possible role in their formation and inhibition, respectively. Correlations with the anthropometric and biochemical variables showed that secondary bile acids could have a role in the amelioration of the glucose and HDL-cholesterol levels. Thus, we have observed a possible relationship between the interaction of the bile acids pool metabolized by the gut microbiota in the metabolic improvements obtained by bariatric surgery in the frame of morbid obesity, deserving further investigation in greater cohorts to decipher the role of each bile acid in the homeostasis of the host for their possible use in the development of microbiota-based therapeutics, such as new drugs, postbiotics or probiotics.

## 1. Introduction

Obesity is a worldwide problem that has reached numbers of pandemic. Obesity is the basis for many other comorbidities such as insulin resistance, hypertension or even trauma problems that trigger a worsening of the quality of life of the patients. Especially worrying is the extreme or morbid obesity, meaning a body mass index (BMI) equal or higher than 40 kg/m^2^ or 35 kg/m^2^ with comorbidities. In this case, the only long-lasting treatment for weight loss is bariatric surgery [1]. The field of gastric surgery includes different procedures, which consider different aspects that must be adapted to the characteristics of the patient in order to increase weight loss and the improvement of other metabolic variables. The two procedures more widely used are Roux-en-Y gastric bypass (RYGB) or sleeve gastrectomy (SG) [2]. These two bariatric surgeries are differentiated in the anatomic rearrangements of the digestive system. In RYGB, the stomach is divided generating a small gastric pouch, which is then anastomosed with the mid-jejunum, creating the Roux or alimentary limb; while in SG a tube-like new stomach is created because of the transection along the greater curvature of the stomach removing the fundus and body [3].

In spite of their great results in terms of weight loss, the complete understanding of how bariatric surgery achieves its improvements is not well documented, although with different pathways and crosstalk between organs involved. In this manner, the gut microbiome, the virtual organ composed by trillions of microorganisms within the intestine, has been introduced as one of those organs with a role in these enhancements [4]. Gut microbiome interacts with its host through different aspects such as parts of its structure, for example the lipopolysaccharide (LPS), or through the metabolites that produces.

Bile acids are being recognized as contributors to many metabolic pathways [5]. One type of these metabolites are the secondary bile acids which gut microbiota are able to chemically modify from the host-derived primary bile acids when they enter the gastrointestinal tract [6]. Secondary bile acids are potent metabolic signals. Host metabolism can be affected by both microbial modifications of bile acids, which leads to altered signaling via bile acid receptors, and by alterations in the composition of the microbiota [7].

Bariatric surgery with its drastic changes over the digestive system profoundly affects gut microbiome which may influence in the weight loss and metabolic improvements reported after bariatric surgery. In order to address this question, our group previously reported that gut microbiome restructuration after two different bariatric surgery procedures, namely RYGB or SG, was different according to the method used [8]. One hypothesis is that gut microbiota alteration after bariatric surgery is driven by changes in the bile acids pool [9]. Primary bile acids amount and profile change depending on the digestive tract rearrangements produced in the bariatric surgery as well as the reduction in the food ingestion.

The current study complements the previous report [8] with the untargeted analysis of the stool metabolome of a subsample of those patients before and after the two bariatric surgery procedures in order to investigate the changes suffered by the bile acids pool with the two surgery methods as well as the relationship with the gut microbiome profile, its function and the metabolic outcomes.

## 2. Results

### 2.1. Clinical Data of the Volunteers

Anthropometric and biochemical data of the volunteers operated by RYGB or SG are shown in Table 1 at the two sampled times. No statistically significant differences were found at baseline or post-surgery between surgeries, although interesting changes were observed within each procedure. As expected, anthropometrical variables significantly changed within each procedure. Main differences appeared in biochemical variables. Although both procedures showed relevant changes in the most of the measured variables, indicating a general improvement of the metabolism after the respective bariatric surgeries, RYGB showed more statistically significant changes in glucose and lipid metabolisms than SG.

### 2.2. Gut Microbiota Results

Although gut microbiota populations of the volunteers after their respective bariatric surgeries, RYGB or SG, did not differ according to the presence/absence of the diverse ASVs (unweighted Unifrac distances, PERMANOVA, *q* = 0.639), the difference was apparent when their abundances were considered (weighted Unifrac distances, PERMANOVA, *q* = 0.033). When changes were taking into account with the longitudinal assessment, changes of the microbiota population did not reach significance but the changes in their distances were more pronounced in the quantitative approach (Supplementary Figure S1a). Alpha diversity indexes did not show any significance (Supplementary Figure S1b).

### 2.3. Microbial Differential Abundance Analysis

**Figure 1:**
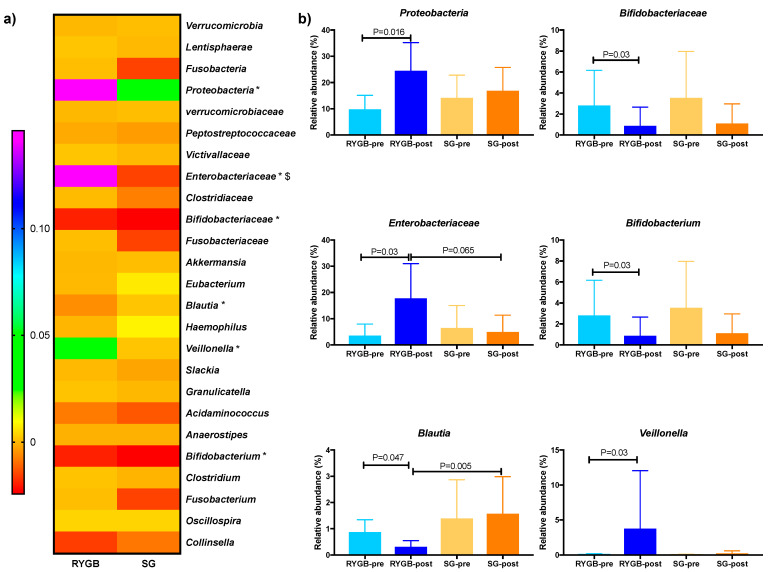
(**a**) Heatmap of the changes in the relative abundance of the microbiota profile at Phylum, Family and Genus levels of the two bariatric surgery procedures tested, Roux-en-Y gastric bypass (RYGB) and sleeve gastrectomy (SG). Differences in percentage are shown. * Indicates changes within the RYGB procedure, $ indicates changes between procedures. (**b**) Bar plots of the statistically significant bacteria from the previous heatmap.

In order to look for the main changes observed between the two procedures of bariatric surgery, a differential abundance analysis was performed over the main bacteria changes observed in our previous report [8] with the samples used in the current report. Although a low number of samples per group was analyzed, the RYGB procedure resulted in the highest changes, with an increase of the *Proteobacteria* phylum (*q* = 0.018), its family *Enterobacteriaceae* (*q* = 0.028), as well as its genus *Veillonella* (*q* = 0.028). The genus *Blautia*, from *Clostridiales*, decreased (*q* = 0.042), in the same manner that the family *Bifidobacteriaceae* (*q* = 0.028) and its genus *Bifidobacterium* (*p* = 0.028) that were also affected in this procedure. However, no significant changes were observed within SG. Finally, only the family *Enterobacteriaceae* (*p* = 0.016) changed in a statistically significant manner between the two types of bariatric surgery (Figure 1).

### 2.4. Bile Acids Profile

**Figure 2:**
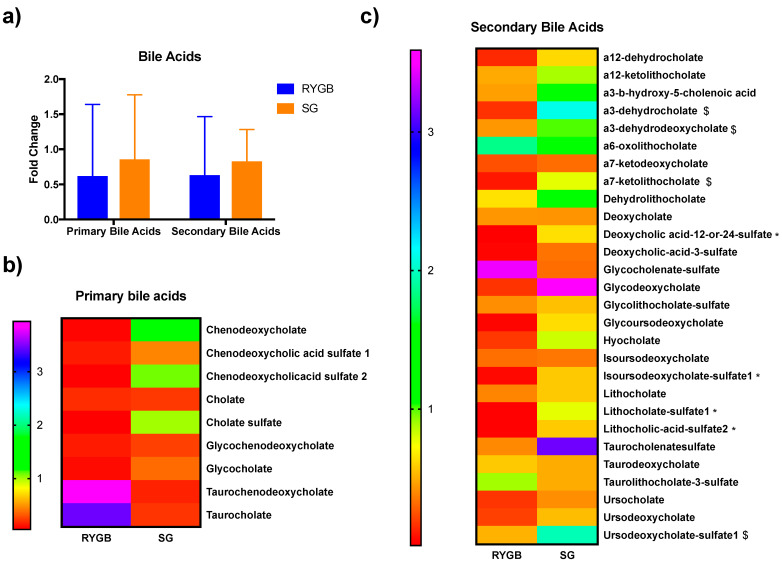
Heatmap of the bile acids fold changes in the two bariatric surgery procedures studies. (**a**) Total bile acids changes. (**b**) Primary bile acids changes. (**c**) Secondary bile acids changes. Changes are shown as fold changes within each procedure; * indicates statistical differences (*p* < 0.05) between procedures, $ indicates a statistical tendency (*p* < 0.10) between procedures.

In general terms, bile acids abundances were reduced with the two procedures of bariatric surgery, primary and secondary bile acids. Although without a statistic significance, RYGB suffered the highest reduction (Figure 2a).

Deeping into the changes of primary bile acids, fold changes are represented in the heatmap of Figure 2b. No primary bile acids changes were statistically significant between procedures. However, observing fold change trends in the figure, it can be inferred that there is a reduction in the most of the primary bile acids with RYGB, with the exception of taurochenodeoxycholate and taurocholate. In SG, the primary bile acids that seem to be increased were chenodeoxycholate, chenodeoxycholic sulfate 2 and cholate sulfate.

About the secondary bile acids, in a general manner, were reduced in the RYGB while increased in SG. The following secondary bile acids fold changes differed between groups (*p* < 0.05): deoxycholic_acid_12_or_24_sulfate, isoursodeoxycholate sulfate 1 and Lithocholate-sulfate1 and Lithocholic-acid-sulfate2, with the next ones showed a tendency (*p* < 0.1): a3-dehydrocholate, a3-dehydrodeoxycholate, a7-ketolithocholate, and Ursodeoxycholate-sulfate1 (Figure 2c, Supplementary Figure S2). 

### 2.5. Relationship between Bacteria and Bile Acid Changes

**Figure 3:**
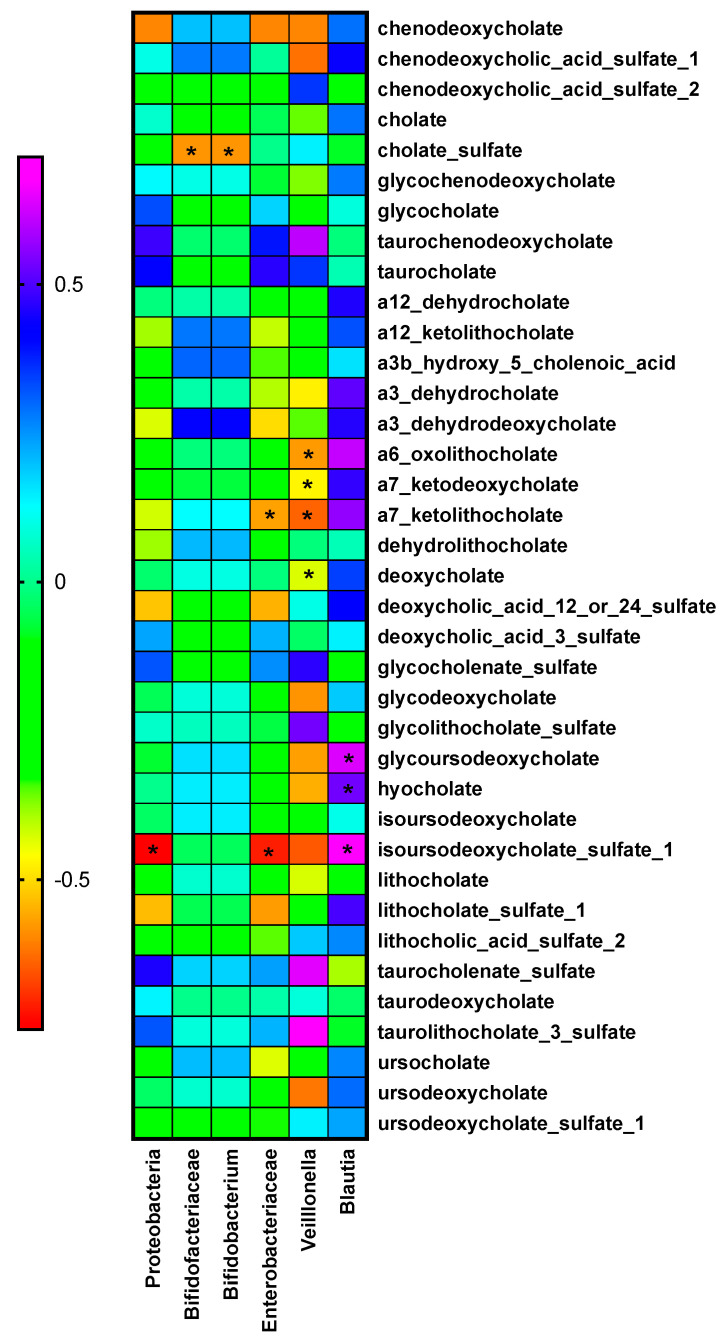
Heatmap of the Spearman correlations between bacteria of interest and secondary bile acids. * Indicates *p* < 0.05.

When changes of bacteria were correlated with the changes in bile acids in the whole population, interesting results emerged. Figure 3 and Supplementary Figure S3 showed the Spearman correlations. The bacteria that showed the main relationship with the bile acids were *Blautia* and *Veillonella*, although not with the same bile acids. In fact, although not significant, the trends of these correlations with these two bacteria were in the inverse manner.

### 2.6. Microbiota Metabolic Pathways

**Figure 4:**
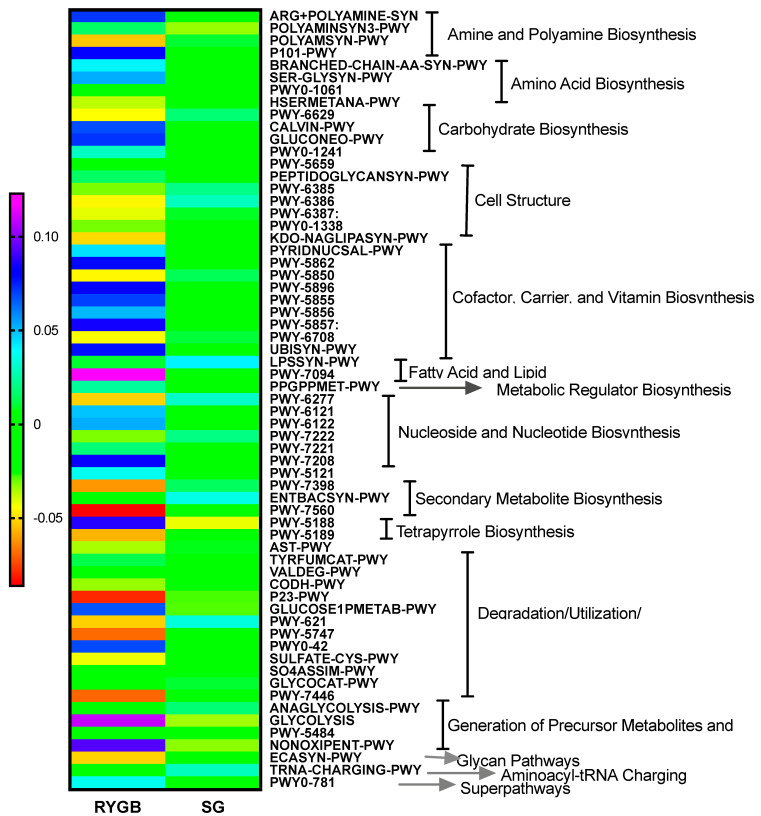
Heatmap of the changes in the metabolic pathways inferred by a Picrust analysis of the 16S rRNA sequences. Metacyc pathways of interest are depicted, as well as their super-classes of entity. Additional information about each pathway-ID can be found in the Supplementary Table S1 in Supplementary Materials.

Bile salt hydrolase (BSH) is the main enzyme that bacteria use for the metabolization of the primary bile acids [10]. Thus, by using PiCRUSt2 we inferred the metabolic pathways of our microbiota populations. We observed a greater amount of this enzyme in SG, although without significance (data not shown). On the other hand, Figure 4 shows the significant changes between procedures. It can be observed the different patterns between RYGB y SG. While SG changes are minimal, RYGB is characterized for more pronounced changes. The main pathways affected are those relative to biosynthesis of different compounds. Interestingly, RYGB seems to have increased pathways of vitamin and polyamines.

### 2.7. Relationship between Biochemical and Anthropometric Variables and Bile Acid Changes

**Figure 5:**
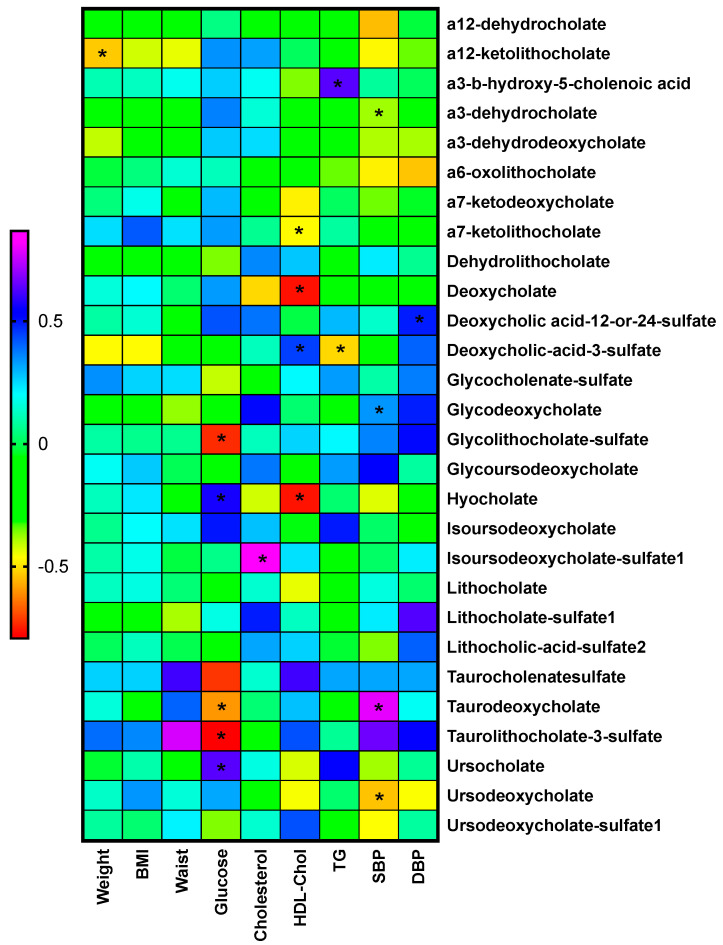
Heatmap of the Spearman correlations between changes in anthropometric and biochemical variables with the fold-changes in secondary bile acids. * Indicates *p* < 0.05.

Figure 5 and Supplementary Figure S4 depicts the correlation analysis between the anthropometric and biochemical variables and bile acids showed in a heatmap. Glucose and HDL-cholesterol were the two variables with the highest relationship with secondary bile acids. Contrary to what was expected, only one secondary bile acid, a12-ketolithocholate, showed a relation with anthropometric variables, in this case, weight.

## 3. Discussion

Bariatric surgery is the most effective strategy to weight loss in the long term. Although its boundaries are well known, the exact mechanisms driving its effects are not well understood. In the current study, we have shown that different procedures of bariatric surgery resulted in different changes in the gut microbiota profile and functionality, as well as in different changes in the primary and secondary bile acids pool triggering in different relationships with the metabolism of the host.

In recent years, gut microbiota has been proposed as one of these mediators, and specially through its participation in the metabolism of bile acids. The relationship between gut microbiota and bile acids is intriguing and in both directions: bile acids are able to modulate the gut microbiota profile and vice versa, the pool of bile salts is shaped by bacterial metabolism. However, this interaction is mainly focused on the 5% of bile acids that escape from the reabsorption by the intestinal cells and arrive in the colon where they enter in close contact with gut microbiota producing the secondary bile acids. In fact, intestinal bacteria use bile salts as environmental signals and in certain cases as nutrients and electron acceptors [11]. In the current study, we have observed that the different rearrangements suffered by the digestive tract in bariatric surgery trigger different profile of gut microbiota, its metabolic capabilities as well as the bile acid pool.

Nowadays, the two bariatric surgery procedures most widely used for the alleviation of extreme obesity are RYGB and SG. These techniques are usually known as examples of malabsorptive-restrictive and restrictive techniques. As it has been previously related in the literature, the more interventionist nature of RYGB provokes bigger changes both in the gut microbiota profile [8] and in the bile acids pool [12]. Similar to our previous report, in this study, we have observed that although gut microbiota populations are formed by similar features, their abundances differed between procedures. These changes seemed to be related to differences driven by members of the families *Enterobacteriaceae* and *Bifidobacteriaceae* [8,13].

The amount and type of bile reaching the intestine can alter gut population due to their antibacterial effect. Moreover, a low level of bile salts favors proliferation of Gram-negative bacteria, while high levels of bile salts favor the proliferation of Gram-positive bacteria [14]. In the current study, we have observed a higher level of primary and secondary bile acids in SG operated individuals. This could be related to the fact that the Gram-negative bacteria *Enterobacteriaceae* are increased in RYGB, while the Gram-positive bacteria *Bifidobacteriaceae* were reduced. Moreover, these are two families known to have the potential capacity to metabolize bile acids. *Bifidobacteriaceae* are bacteria with a high BSH capacity, while *Enterobacteriaceae* lacks this capacity [15] but are able to transform bile acids through dehydroxylation [6], although we have not observed differences in BSH capacity between procedures.

In the present study, we have observed that the bile acid pool is affected in a different manner depending on the bariatric surgery procedure, being the RYGB the one with the highest changes, because of the re-routing of the bile flow to the mid-jejunum. The immense majority of fecal bile acids were reduced after RYGB. It is worth noting that many studies have reported an increase of bile acids after bariatric surgery, although in serum [16], possibly implicating a higher absorption through the blood, but something not measured in this study. The main differences observed in the bile acids pool between procedures were the relatives to the primary bile acid cholate, one of the two major bile salts in the liver together with chenodeoxycholic acid. When changes in bacteria were related to the changes in bile acids, most of the relationships resulted from *Veillonella* and *Blautia*, with no relation with *Bifidobacterium*. This could be because of a preference for *Bifidobacterium* for conjugated bile acids [17].

*Veillonella* was increased after the RYGB. *Veillonella* is a commensal known for its lactate-degrading and performance enhancing properties that has been previously related to bile acids because of its growth when the drug aldafermin, an analog of FGF19 that regulates the bile acids synthesis, was administered [18]. These authors stated that *Veillonella* growth was promoted because of the suppression of toxic bile acids synthesis. In the current study, this result could be related to the diminishing of bile acids found after the RYGB. About *Blautia*, this bacterium is closely related to human bile acid 7α-dehydroxylating species [19]. It has been shown that 7α-dehydroxylation from primary to secondary BAs provides an energy advantage to the bacteria [6].

At the same time, bile acids are potent signaling molecules of the host homeostasis as well as the own microbiota population. Bile acids are signaling molecules that activate nuclear farnesoid X receptor (FXR) and membrane G protein-coupled bile acid receptor−1 (Gpbar-1, also known as TGR5) to maintain metabolic homeostasis and protect liver and other tissues and cells from bile acid toxicity. This axis has been recognized as a mediator for the metabolic improvements after bariatric surgery [20]. In fact, the transfer of gut microbiota from RYGB-treated mice to germ-free mice resulted in weight loss and decreased fat mass in the recipient animals [21]. Changes in BA physiology and receptor activities after RYGB and SG likely support weight loss and promote sustained metabolic improvements [22]. In this study, most of the relationships between bile acids and anthropometric and biochemical variables have been observed with glucose and HDL-cholesterol. Some data suggest that the altered enterohepatic circulation of BAs could contribute to improved insulin sensitivity and cholesterol metabolism following bariatric surgery [23]. In line with these changes, and although changes in the microbiota population observed in this study could seem minor, the PICRUSt2-imputed metagenomic functional analysis showed the profound changes suffered in the metabolic capacities of these populations with a clear differentiation between procedures, being SG the most conservative technique and RYGB suffering changes toward biosynthesis pathways of polyamines, cofactor and vitamins as well as carbohydrates. These trends may suggest a possible role of gut microbiota in the readjustments of metabolism after bariatric surgery.

This study encourages a better understanding of the interactions between bacteria and bile salts, something that may inspire novel therapeutic strategies. This kind of secondary bile acid is a promising and safe class of drugs for the treatment of different metabolic diseases. In fact, the secondary bile acid ursodeoxycholic acid (UDCA) is already an FDA-approved drug for cholestatic liver disorders. Interestingly, administration of UDCA, tauroursodeoxycholic acid (TUDCA), or glycoursodeoxycholic acid (GUDCA) prevented the loss of *Clostridium* cluster XIVa and increased the abundance of *Akkermansia muciniphila* [24]. In the current study, GUDCA and other relatives to UDCA have been also related to *Blautia* where their levels were positively correlated, possibly indicating a role in its production, while *Veillonella* levels were inversely correlated indicating a possible inhibition role of this bacteria by these bile acids, which deserves further studies.

Although the results presented in the current study are promising, we are aware of its limitations due to the low sample size and its correlative nature. However, this proof-of-concept assay lays the foundations of future research delving into the relationships between gut microbiota and bile acids in powerful clinical trials to be able to establish particular roles in the homeostasis of patients suffering of metabolic diseases.

## 4. Material and Methods

The study was assessed in 16 patients suffering morbid obesity who underwent bariatric surgery, 8 RYGB and 8 SG, between May 2015 and March 2017 and who accepted to enter the study. Fecal samples were collected prior intervention (baseline) and 3 months after the correspondent procedure. Volunteers were instructed to follow a hypocaloric Mediterranean diet with a 600 Kcal/day of caloric deficit, according to the Harris-Benedict equation. After surgery, subjects started with a liquid diet for 1–2 weeks, followed by crushed or semi-soft diet for 2 weeks. In the next following weeks post-surgery, solid diet was introduced progressively. During all these weeks, patients received protein supplementation to prevent protein malnutrition. The Biomedical Research Ethic Coordinator Committee of Andalucía (CCEIBA) was approved on 20 December 2016 under the code CP1600163.

### 4.1. Anthropometric and Laboratory Measurements

Standardized procedures were used for the measurements of weight and height and body mass index (BMI) was calculated as weight (kg)/height^2^ (m^2^).

Blood samples were collected after an overnight fast at the different study point. The serum was separated and immediately stored at −80 °C until posterior analysis. Enzimatic methods were used for the measurement of the levels of cholesterol, triglycerides, high density lipoprotein cholesterol (HDL-cholesterol), and glucose (Randox Laboratories Ltd., Crumlin, UK). The Friedewald formula was used for the calculation of the levels of the low-density lipoprotein (LDL) cholesterol [25]. Glycosylated hemoglobin (HbA1c) was determined by Dimension Vista autoanalyzer (Siemens Healthcare Diagnostics, Munich, Germany).

The homeostasis model assessment of insulin resistance (HOMA-IR) index was calculated according to the formula: HOMA-IR = fasting plasma insulin (µU/mL) × fasting plasma glucose (mmol/L)/22.5.

### 4.2. Fecal Samples Analysis

Fecal samples were obtained by the volunteers and stored at −80 °C for subsequent analysis.

DNA for the subsequent microbiota analysis was extracted from 200 mg of fecal samples with the QIAamp DNA stool Mini kit (Qiagen, Hilden, Germany) according to the manufacturer’s protocols. A Nanodrop spectrophotometer (Nanodrop Technologies, Wilmington, DE, USA) was used for the measurement of the DNA concentration at 260 nm, and the A260/A280 ratio for purity verification.

### 4.3. Gut Microbiota Analysis

The Ion 16S Metagenomics kit was used to build the sequencing libraries from the 16S rRNA gene and posteriorly templated on the automated Ion Chef system followed by sequencing on an Ion S5 (everything from Thermo Fisher Scientific, Waltham, MA, USA).

### 4.4. Sequence Data and Statistical Analysis

The open-source Quantitative Insights into Microbial Ecology (QIIME2, version 2019.10) software was used to analyze the generated sequences [26]. These quality sequences were further translated into amplicon sequence variants (ASVs) using DADA2 with adapted parameters for Ion Torrent data [27]. The diversity plugin was used for diversity analysis. α-diversity was assessed through four different indexes (Shannon, Faith_pd, Pielou and observed ASVs), while β-diversity was measured using UniFrac distances in its unweighted and weighted versions. Taxonomic analysis was assessed through the sequences clustering with *vsearch* [28] and the reference base Greengenes version 13_8 at 97% of identity.

Phylogenetic Investigation of Communities by Reconstruction of Unobserved States plugin (PICRUSt2) [29] was used to predict metagenome function within QIIME2. MetaCyc pathways [30] were normalized within QIIME2 and further analyzed with STAMP [31].

Longitudinal plugin was used for the further differential abundance analysis of the bacteria as well as metabolic pathways found of interest.

### 4.5. Metabolomics Analysis

Fecal metabolome analysis was performed by Metabolon Inc. Briefly, following receipt samples were maintained at −80 °C until processed. Samples were prepared using the automated MicroLab STAR^®^ system (Hamilton Company). Samples were analyzed by Ultrahigh Performance Liquid Chromatography-Tandem Mass Spectroscopy (UPLC-MS/MS). All methods utilized a Waters ACQUITY ultra-performance liquid chromatography (UPLC) and a Q-Exactive high resolution/accurate mass spectrometer (Thermo Scientific) interfaced with a heated electrospray ionization (HESI-II) source and Orbitrap mass analyzer operated at 35,000 mass resolution. Samples were dried and reconstituted in solvents according to each method. The Mass Spectroscopy analysis alternated between Mass Spectroscopy and data-dependent MSn scans using dynamic exclusion. The scan range covered 70–1000 m/z. Bioinformatics analysis consisted of four major components, listed here: Laboratory Information Management System (LIMS), the data extraction and peak-identification software, data processing tools for QC and compound identification, and a collection of information interpretation and visualization tools through the LAN backbone, and a database server running Oracle 10.2.0.1 Enterprise Edition. Data were curated, metabolites quantified using the area-under-the-curve method, and data normalized for posterior statistical analysis.

### 4.6. Statistical Analysis

Statistical software package SPSS version 22.0 (SPSS Inc., Chicago, IL, USA) was used to study differences in anthropometric and biochemical variables. Differences between the groups were analyzed by a one-way ANOVA followed by a Duncan’s post hoc test and Wilcoxon signed-rank test was used to calculate differences between baseline and the end of the intervention. Relationships were tested by Spearman correlation. Values were considered statistically significant when *p* or *q* value < 0.05.

## 5. Conclusions

In sum, we have observed a clear relationship between some particular microbiota members related to changes with bariatric surgery, with changes in the bile acid pool. *Veillonella* and *Blautia* are the main features that seem to participate in this process. The correlative analysis could indicate that *Blautia* could enhance some important secondary bile acids such as those of the ursodeoxycholate family. Moreover, these bile acids changes have been related to changes in the glucose and lipid metabolism, especially to glucose and HDL-cholesterol levels, suggesting a possible role of these secondary bile acids formed by these particular members of the gut microbiota in the amelioration of the metabolism after a bariatric surgery. These pilot study results deserve to continue studying these relationships in order to advance in the use of microbial metabolites for the development of secondary bile acids therapeutics or for the development of probiotics that enhance the production of these particular metabolites to fight against metabolic disorders such as obesity.

## Figures and Tables

**Table 1:** Biochemical and clinical characteristics of both study groups at pre-and post-surgery timepoints.

	RYGB		SG	
	Pre-Surgery	Post-Surgery	Pre-Surgery	Post-Surgery
Gender (M/F)	3/5		4/4	
Age, years	47.12 ± 8.02		46.75 ± 12.71	
Weight, kg	116.85 ± 17.03	96.66 ± 16.16 *	127.01 ± 24.90	104.21 ± 15.84 *
BMI, kg/m^2^	43.87 ± 6,58	36.16 ± 5.64 *	45.49 ± 4.99	37.64 ± 4.65 *
Waist, cm	131.63 ± 13,05	115.25 ± 10.31 *	135.00 ± 11.82	118.71 ± 9.98 *
Hip, cm	133.88 ± 15.08	121.75 ± 13.85 *	136.94 ± 10.66	123.71 ± 10.63 *
HbA1C, %	7.49 ± 1.77	5.53 ± 0,26 *	6.38 ± 1.43	5.55 ± 0.56 *
HOMA-IR	7.33 ± 3.50	2.81 ± 2.11 *	9.20 ± 6.69	3.00 ± 1.91
Insulin, mU/mL	22.28 ± 11.17	11.61 ± 7.17 *	33.29 ± 24.29	14.72 ± 5.70 *
Glucose, mg/dL	130.13 ± 38.88	93.86 ± 9.26 *	108.88 ± 38.16	85.71 ± 14.30
SBP, mmHg	136.63 ± 11.33	127.33 ± 20.39	144.50 ± 22.42	141.38 ± 16.12
DBP, mmHg	80.00 ± 8.33	75.00 ± 9.34	85.88 ± 5.38	85.25 ± 6.50
Cholesterol, mg/dL	196.88 ± 39.94	151.71 ± 36.70 *	184.88 ± 28.39	174.43 ± 34.80
HDL-chol, mg/dL	45.00 ± 16.89	41.86 ± 11.45	42.63 ± 8.40	41.29 ± 11.25
LDL-chol, mg/dL	114.68 ± 29.95	86.63 ± 34.59	112.31 ± 20.45	111.51 ± 26.04
TG, mg/dL	186.00 ± 82.50	116.14 ± 48.13 *	228.75 ± 276.57	108.14 ± 38.32
GOT, U/L	27.25 ± 11.82	21.14 ± 7.22	23.25 ± 8.12	17.71 ± 4.85
GPT, U/L	42.25 ± 19.03	28.71 ± 9.41	30.75 ± 17.30	20.83 ± 5.15 *
GGT, U/L	48.50 ± 7.78	21.57 ± 12.53	36.33 ± 13.01	20.86 ± 7.99

RYGB = Roux-en-Y gastric bypass; SG = sleeve gastrectomy; BMI = body mass index; HBA1C = glycated hemoglobin; HOMA-IR = homeostatic model assessment of insulin resistance; SBP = systolic blood pressure; DBP = diastolic blood pressure; HDL-chol = high-density lipoprotein cholesterol; LDL-chol = low-density lipoprotein cholesterol; TG = triglycerides; GOT = glutamic oxaloacetic transaminase; GPT = glutamic pyruvic transaminase; GGT = gamma-glutamyl transferase. Values are presented as means ± standard deviation. * Values are significantly different within the same study group for *p* = 0.05.

## Data Availability

Not applicable.

## Supplementary Materials

The following are available online at https://www.mdpi.com/article/10.3390/metabo11110733/s1, Figure S1: Gut microbiota diversity measurements, Figure S2: Bar graphs of the bile acids fold changes in the two bariatric surgery procedures studies, Figure S3: Scatterplots of Spearman correlations between changes in bacteria relative abundance and fold change, Figure S4: Scatterplots of the Spearman correlations between changes in anthropometric and biochemical variables with the fold-changes, Table S1: List of the pathways.
