# Supplementary material for: Gut Microbiota Metabolism of Bile Acids Could Contribute to the Bariatric Surgery Improvements in Extreme Obesity
Source: Metabolites. 2021 Oct 27;11(11):733. doi: 10.3390/metabo11110733 (PMC8620296; doi:10.3390/metabo11110733)

Supplementary Table S1. List of the pathways.

BioCyc Pathway ID	Pathway
ARG+POLYAMINE-SYN	superpathway of arginine and polyamine biosynthesis
POLYAMINSYN3-PWY	superpathway of polyamine biosynthesis II
POLYAMSYN-PWY	superpathway of polyamine biosynthesis I
P101-PWY	ectoine biosynthesis
BRANCHED-CHAIN-AA-SYN-PWY	superpathway of branched chain amino acid biosynthesis
SER-GLYSYN-PWY	superpathway of L-serine and glycine biosynthesis I
PWY0-1061	superpathway of L-alanine biosynthesis
HSERMETANA-PWY	L-methionine biosynthesis III
PWY-6629	superpathway of L-tryptophan biosynthesis
CALVIN-PWY	Calvin-Benson-Bassham cycle
GLUCONEO-PWY	gluconeogenesis I
PWY0-1241	ADP-L-glycero-&beta
PWY-5659	GDP-mannose biosynthesis
PEPTIDOGLYCANSYN-PWY	peptidoglycan biosynthesis I (meso-diaminopimelate containing)
PWY-6385	peptidoglycan biosynthesis III (mycobacteria)
PWY-6386	UDP-<i>N</i>-acetylmuramoyl-pentapeptide biosynthesis II (lysine-containing)
PWY-6387	UDP-N-acetylmuramoyl-pentapeptide biosynthesis I
PWY0-1338	polymyxin resistance
KDO-NAGLIPASYN-PWY	superpathway of (Kdo)2-lipid A biosynthesis
PYRIDNUCSAL-PWY	NAD salvage pathway I (PNC VI cycle)
PWY-5862	superpathway of demethylmenaquinol-9 biosynthesis
PWY-5850	superpathway of menaquinol-6 biosynthesis
PWY-5896	superpathway of menaquinol-10 biosynthesis
PWY-5855	ubiquinol-7 biosynthesis (early decarboxylation)
PWY-5856	ubiquinol-9 biosynthesis (early decarboxylation)
PWY-5857	ubiquinol-10 biosynthesis (early decarboxylation)
PWY-6708	ubiquinol-8 biosynthesis (early decarboxylation)
UBISYN-PWY	superpathway of ubiquinol-8 biosynthesis (early decarboxylation)
LPSSYN-PWY	superpathway of lipopolysaccharide biosynthesis
PWY-7094	fatty acid salvage
PPGPPMET-PWY	ppGpp metabolism
PWY-6277	superpathway of 5-aminoimidazole ribonucleotide biosynthesis
PWY-6121	5-aminoimidazole ribonucleotide biosynthesis I
PWY-6122	5-aminoimidazole ribonucleotide biosynthesis II

**PWY-7222**	guanosine deoxyribonucleotides de novo biosynthesis II
**PWY-7221**	guanosine ribonucleotides de novo biosynthesis
**PWY-7208**	superpathway of pyrimidine nucleobases salvage
**PWY-5121**	superpathway of geranylgeranyl diphosphate biosynthesis II (via MEP)
**PWY-7398**	coumarins biosynthesis (engineered)
**ENTBACSYN-PWY**	enterobactin biosynthesis
**PWY-7560**	methylerythritol phosphate pathway II
**PWY-5188**	tetrapyrrole biosynthesis I (from glutamate)
**PWY-5189**	tetrapyrrole biosynthesis II (from glycine)
**AST-PWY**	L-arginine degradation II (AST pathway)
**TYRFUMCAT-PWY**	L-tyrosine degradation I
**VALDEG-PWY**	L-valine degradation I
**CODH-PWY**	reductive acetyl coenzyme A pathway I (homoacetogenic bacteria)
**P23-PWY**	reductive TCA cycle I
**GLUCOSE1PMETAB-PWY**	glucose and glucose-1-phosphate degradation
**PWY-621**	sucrose degradation III (sucrose invertase)
**PWY-5747**	2-methylcitrate cycle II
**PWY0-42**	2-methylcitrate cycle I
**SULFATE-CYS-PWY**	superpathway of sulfate assimilation and cysteine biosynthesis
**SO4ASSIM-PWY**	assimilatory sulfate reduction I
**GLYCOCAT-PWY**	glycogen degradation I
**PWY-7446**	sulfoquinovose degradation I
**ANAGLYCOLYSIS-PWY**	glycolysis III (from glucose)
**GLYCOLYSIS**	glycolysis I (from glucose 6-phosphate)
**PWY-5484**	glycolysis II (from fructose 6-phosphate)
**NONOXIPENT-PWY**	pentose phosphate pathway (non-oxidative branch) I
**ECASYN-PWY**	enterobacterial common antigen biosynthesis
**TRNA-CHARGING-PWY**	tRNA charging
**PWY0-781**	aspartate superpathway

Supplementary Figure S1. Gut microbiota diversity measurements. a) Beta diversity: Unweighted and Weighted Unifrac distances. b) Alpha diversity indexes. It is presented the values at final point (histograms) and changes in values between final and baseline time-points (box and whiskers plots). RYGB is shown in blue; SG is shown in orange.

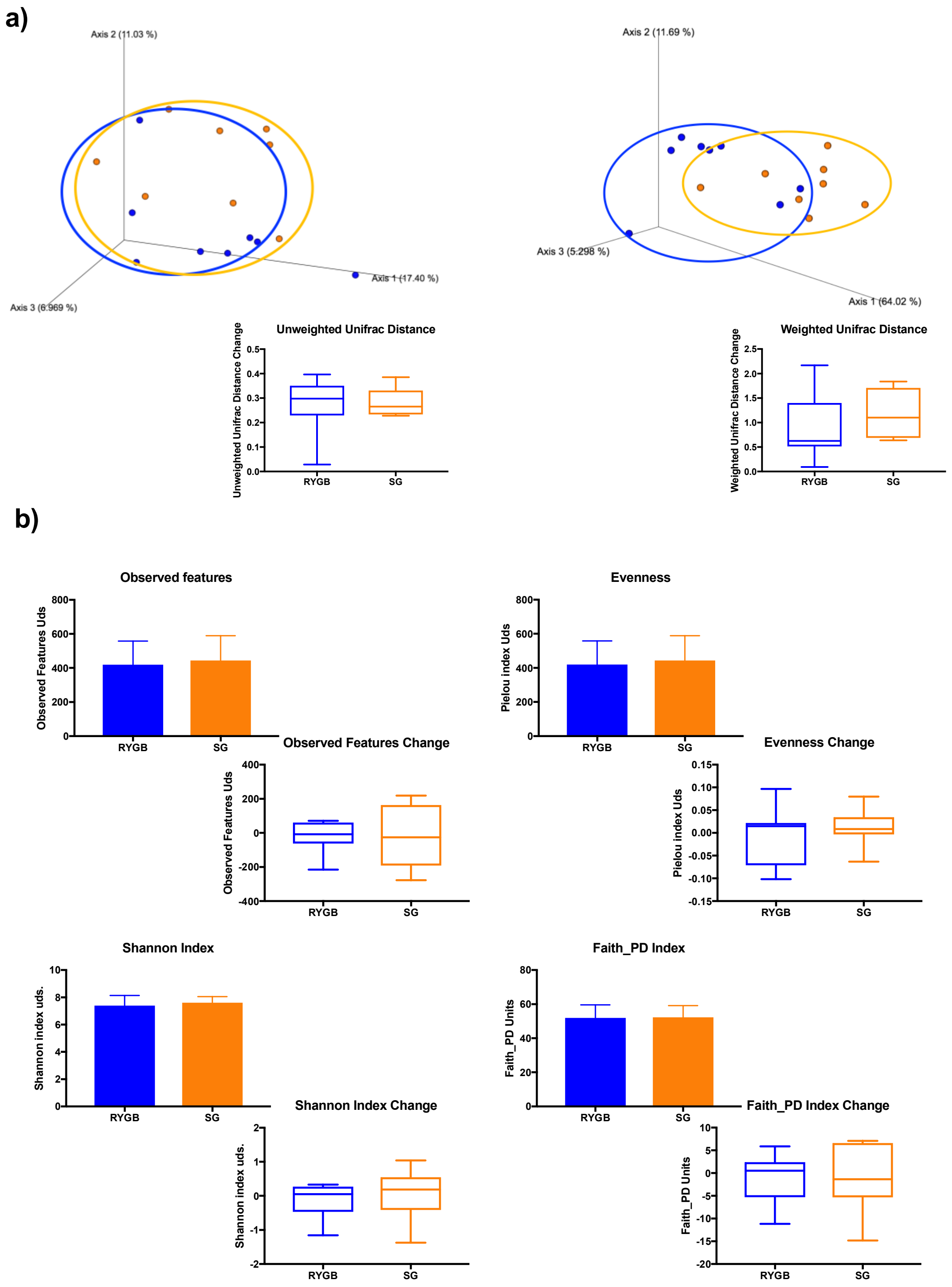

Supplementary Figure S2. Bar graphs of the bile acids fold changes in the two bariatric surgery procedures studies that resulted statistically significant between procedures (P<0.05) or at least with a statistical tendency (P<0.10).

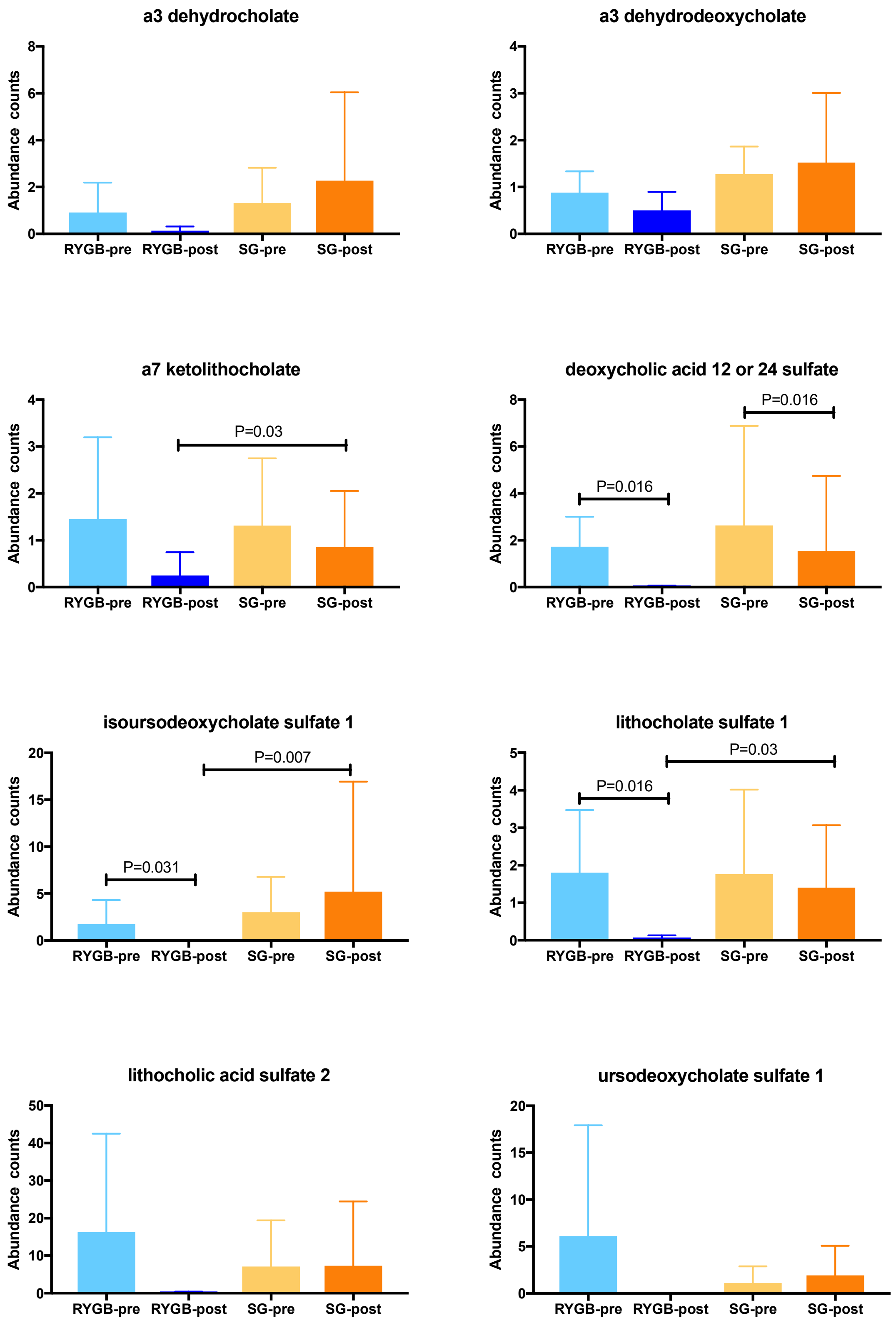

Supplementary Figure S3. Scatterplots of Spearman correlations between changes in bacteria relative abundance and fold change (FC) of the secondary bile acids statistically significant (P<0.05).

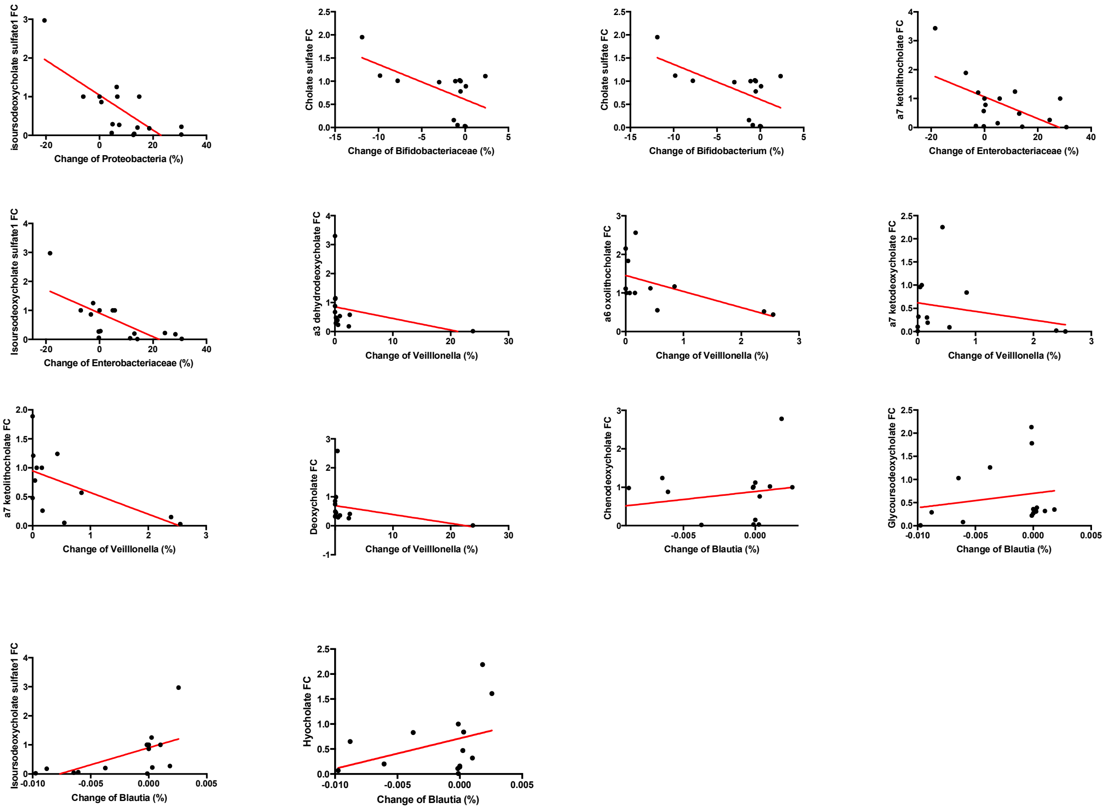

Supplementary Figure S4. Scatterplots of the Spearman correlations between changes in anthropometric and biochemical variables with the fold-changes (FC) in secondary bile acids statistically significant (P<0.05).

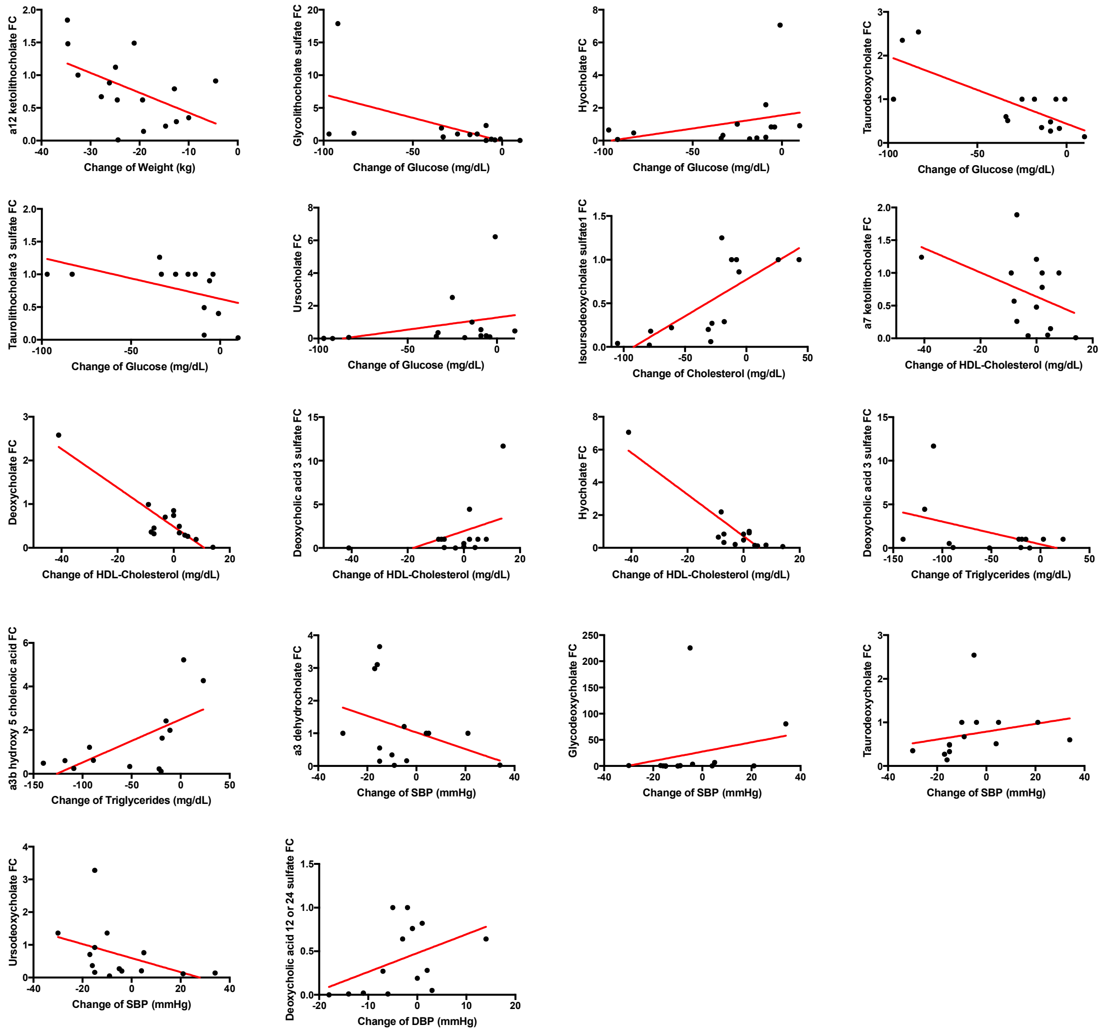

Supplement: Supplementary file 1 [file metabolites-11-00733-s001.zip › metabolites-1407889-SI.pdf]
